# Non-ketotic Hyperglycemic Hemichorea in a Patient Newly Diagnosed With Diabetes: A Rare Neurological Manifestation of Uncontrolled Diabetes

**DOI:** 10.7759/cureus.84067

**Published:** 2025-05-13

**Authors:** Usamah Al-Anbagi, Mohamed Mohamedali, Muayad K Ahmad, Muhammad Sharif, Abdulqadir J Nashwan

**Affiliations:** 1 Internal Medicine, Hamad Medical Corporation, Doha, QAT; 2 Nursing & Midwifery Research, Hamad Medical Corporation, Doha, QAT

**Keywords:** glycosylated hemoglobin (hba1c), hyperintensity in basal ganglia, insulin, magnetic resonance imaging (mri), non-ketotic hyperglycemic hemichorea-hemiballismus (nhh)

## Abstract

Non-ketotic hyperglycemic hemichorea-hemiballismus (NHH) is a rare but reversible movement disorder associated with poorly controlled diabetes mellitus. It is characterized by involuntary, non-suppressible choreiform, ballistic movements or several other involuntary movements (dystonia, tremors, etc), contralateral striatal hyperintensity on MRI, and hyperglycemia without ketosis. The exact pathophysiology remains unclear, but prompt glycemic control is essential for symptom resolution. We report the case of a 41-year-old man with no prior history of diabetes who presented with an acute onset of hemichorea affecting the left upper and lower limbs. Laboratory findings revealed hyperglycemia (18 mmol/L) with an elevated HbA1c (>12%), while brain MRI demonstrated characteristic T1 hyperintensity in the right basal ganglia. The patient was started on insulin therapy and oral antihyperglycemic agents, along with neuroleptics for symptomatic relief. By the third day of treatment, his movements began to improve. This case underscores the importance of recognizing NHH in patients with new-onset movement disorders, emphasizing the role of neuroimaging and early glycemic management for favorable outcomes.

## Introduction

Non-ketotic hyperglycemic hemichorea-hemiballismus (NHH), also referred to as diabetic striatopathy, is a rare but well-recognized neurological complication of diabetes mellitus [1,2]. It is characterized by involuntary, hyperkinetic movements affecting one side of the body, typically contralateral to striatal abnormalities seen on neuroimaging [2]. The estimated prevalence of NHH is approximately one in 100,000, though this may be underestimated due to underreporting and misdiagnosis [3]. While most cases occur in the setting of poorly controlled, longstanding type 2 diabetes, it can also present in newly diagnosed diabetes or cases of acute hyperglycemia, often severe, with glucose levels typically exceeding 400 mg/dL [4]. Risk factors include older age and female sex, with the condition most commonly described in elderly East Asian women, though it may be underdiagnosed in Western populations [5]. Despite its association with hyperglycemia, the exact pathophysiological mechanisms leading to striatal changes and choreiform movements remain unclear.

Clinically, patients with NHH typically present with acute-onset hemichorea involving both the arm and leg, with occasional facial involvement or bilateral symptoms. Newly described dystonia is another commonly missed initial presentation of hyperglycemia-induced involuntary movement [6-8]. MRI findings are crucial in diagnosis, with T1-weighted hyperintensity in the contralateral striatum being the hallmark feature. However, this is not a very sensitive marker; therefore, the absence of T1 hyperintensity (or non-contrast CT hyperdensity) should not exclude the diagnosis if clinical suspicion is high, such as in cases with new-onset hyperglycemia, new involuntary movements, and symptom reversal after glycemic correction [9]. CT scans can show hyperdensity in the affected region but are less sensitive and may appear normal in early or mild cases [10]. Unlike hemorrhagic stroke, which may initially be suspected on CT, NHH is distinguished by normal diffusion-weighted and susceptibility imaging [10]. Symptom resolution generally occurs within days to weeks after glycemic control is achieved, but in some cases, abnormal movements may persist for over a year or recur. Short-term use of symptomatic treatment, such as antipsychotic medications, may be necessary in severe cases. Early recognition and management of NHH are essential to prevent complications and facilitate recovery.

## Case presentation

A 41-year-old male, previously healthy with no significant past medical history, presented with involuntary and purposeless movements on the left side of his body that had begun a few days prior to admission. The movements were continuous, non-suppressible, purposeless, and unrelated to weakness or sensory loss. Notably, the chorea completely settled and disappeared during sleep. He also reported a few days of low-grade fever and a runny nose, which had resolved two days before the onset of the involuntary movements. He denied any recent trauma, headache, dizziness, visual disturbances, or seizures. He denied any history of neurological disorders, strokes, or similar episodes. There was no family history of movement disorders. He denied alcohol use, tobacco use, or other substance abuse.

Clinical examination revealed a temperature of 36.5 °C, a heart rate of 76 beats per minute, a respiratory rate of 18 breaths per minute, high blood pressure of 159/95 mmHg, and oxygen saturation of 99%. Neurological examination showed purposeless involuntary movements of the left upper and lower limbs, with the upper limb being more affected (Video 1). The remainder of the neurological examination was unremarkable. Other systemic examinations were also unremarkable.

**Video 1:** Non-ketotic hyperglycemic hemichorea-hemiballismus in a newly diagnosed diabetic patient Neurological examination showed purposeless involuntary movements of the left upper and lower limbs, with the upper limb being more affected

Initial laboratory investigations revealed a high blood glucose level of 18 mmol/L, with no prior diagnosis of diabetes, and mild hyponatremia (130 mmol/L), likely pseudohyponatremia. Further testing showed a significantly elevated HbA1c of >12%, normal thyroid function tests (TFTs), and a normal anti-streptolysin O (ASO) titer. The pH was 7.4 (normal), and beta-hydroxybutyrate was within normal limits (0.27). Other laboratory values were also within normal limits (Table 1).

**Table 1:** Laboratory investigations ALT: alanine aminotransferase; APTT: activated partial thromboplastin time; AST: aspartate aminotransferase; CRP: C-reactive protein; FT4: free thyroxine; HbA1c: glycated hemoglobin; INR: international normalized ratio; PT: prothrombin time; TSH: thyroid-stimulating hormone

Parameters	Patient values on admission	Patient values at discharge	Reference values
Total leukocytes (x10^3^/uL)	5.8	6	6.2
Hematocrit (%)	44.1	40.1	40
Hemoglobin (gm/dL)	15.1	14	13-17
Platelet (x10^3^/uL)	281	271	150-410
Serum urea (mmol/L)	6.1	6.8	2.5-7.8
Serum creatinine (umol/L)	102	111	62-106
Serum potassium K (mmol/L)	4.3	4.1	3.5-5.3
Serum sodium (mmol/L)	130	136	133-146
Serum calcium (mmol/L)	2.58	2.47	2.2-2.6
Serum phosphorous (mmol/L)	1.48	-	0.8-1.5
Serum magnesium (mmol/L)	0.82	0.7	0.7-1
Serum total protein (gm/L)	78	71	60-80
Serum albumin (gm/L)	37	32	35-50
PH	7.40	7.36	7.320-7.420
Beta-Hydroxybutyrate (mmol/L)	0.27	-	0.03 -0.3
ALT (IU/L)	50	52	0-41
AST (IU/L)	38	29	0-41
Alkaline phosphatase (U/L)	160	146	40–129
Serum total bilirubin (mg/dl)	14	11	0-21
CRP (mg/L)	3.1	<2	0-5
HbA1c (%)	>12	-	<6
TSH (mIU/L)	2.41	-	0.34-4.20
FT4 (pmol/L)	18.7	-	11-23.3
Antistreptolysin O titer (IU/mL)	81	-	0-200
Ceruloplasmin (mg/dL)	27	-	15-30
PT (seconds)	11.2	-	9.4-12.5
INR	1	-	<1
APTT (seconds)	32.2	-	25.1- 36.5
Random blood sugar level (mmol/L)	18	9.9	3.3-7.8

**Figure 1:**
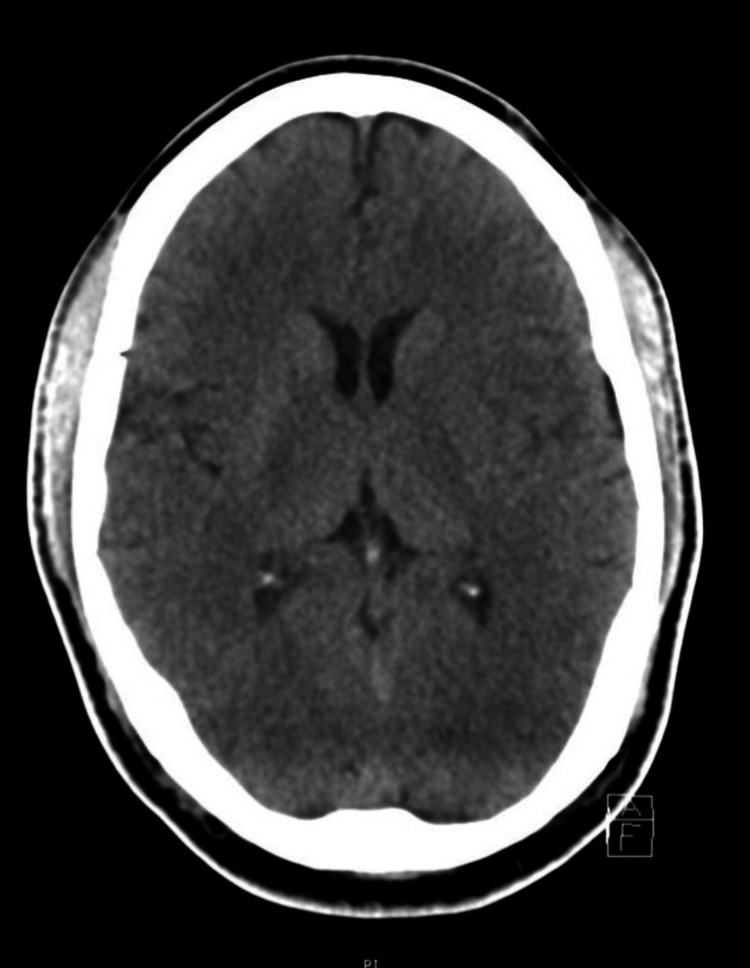
Computed tomography (axial view), unremarkable study

Radiological investigations included an unremarkable CT brain (Figure 1) and a normal chest X-ray.

**Figure 2:**
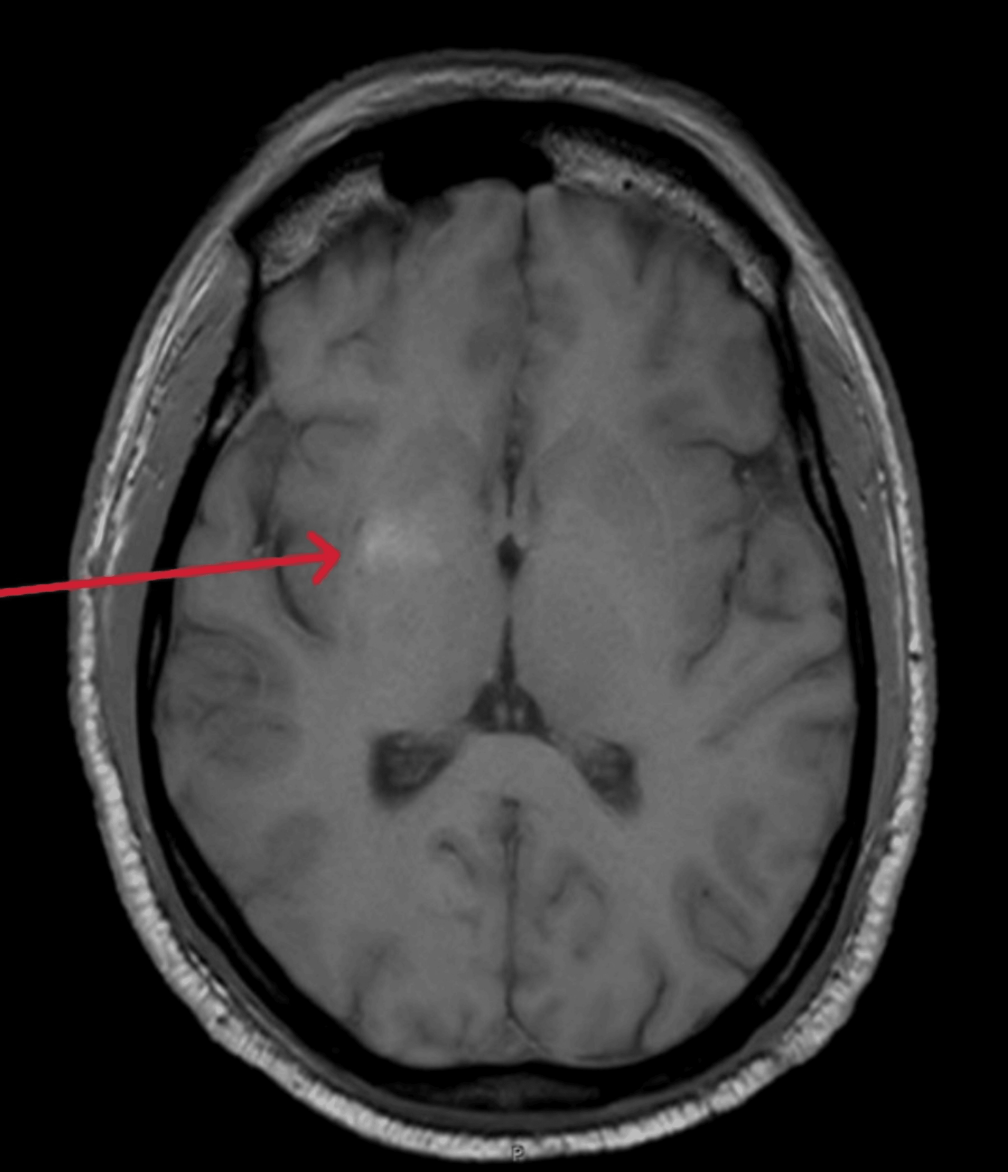
T1-weighted magnetic resonance imaging (axial view) showing hyperintensity noted in the right basal ganglia (red arrow)

**Figure 3:**
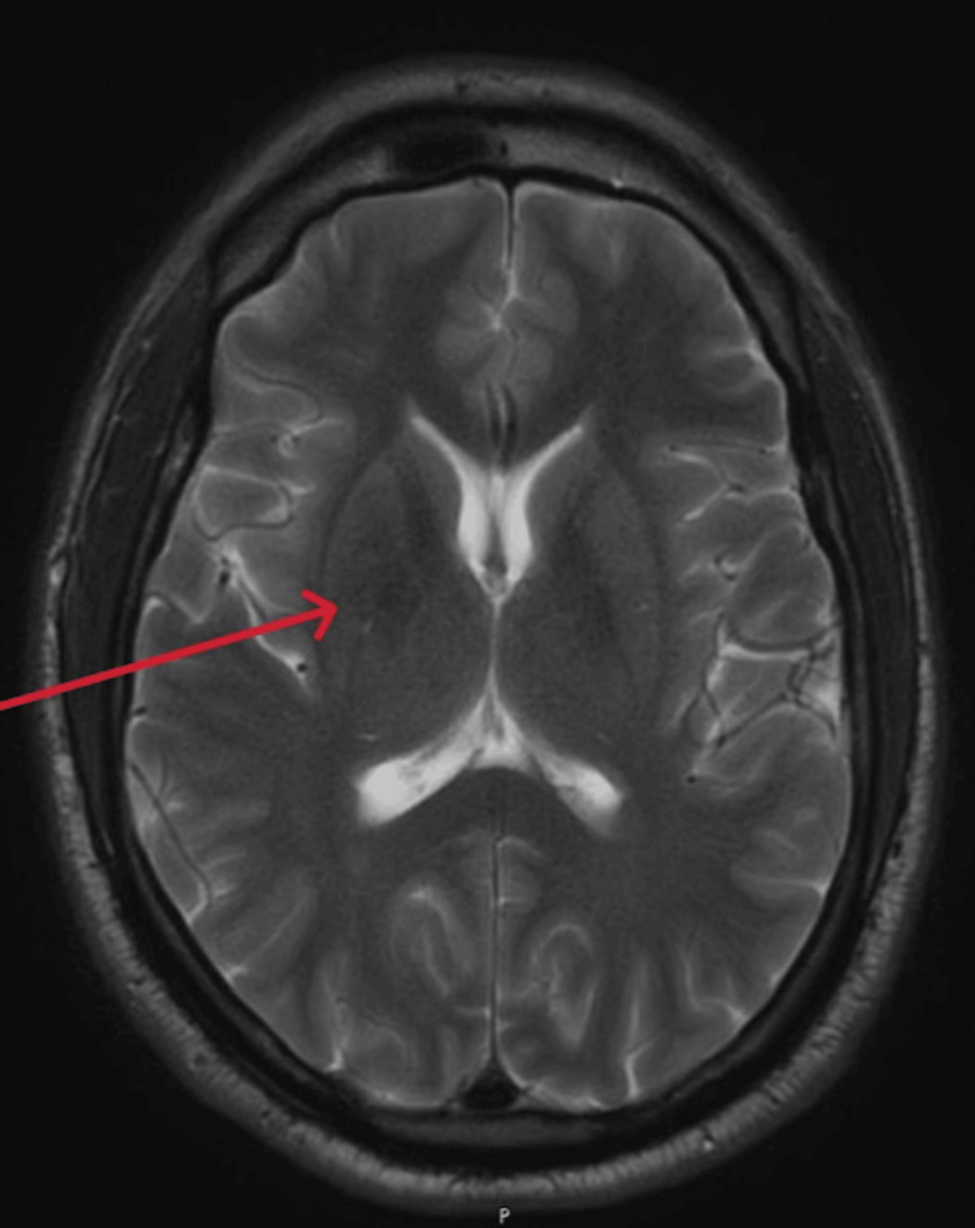
T2-weighted magnetic resonance imaging showing subtle hypointensity noted in the right basal ganglia (red arrow)

**Figure 4:**
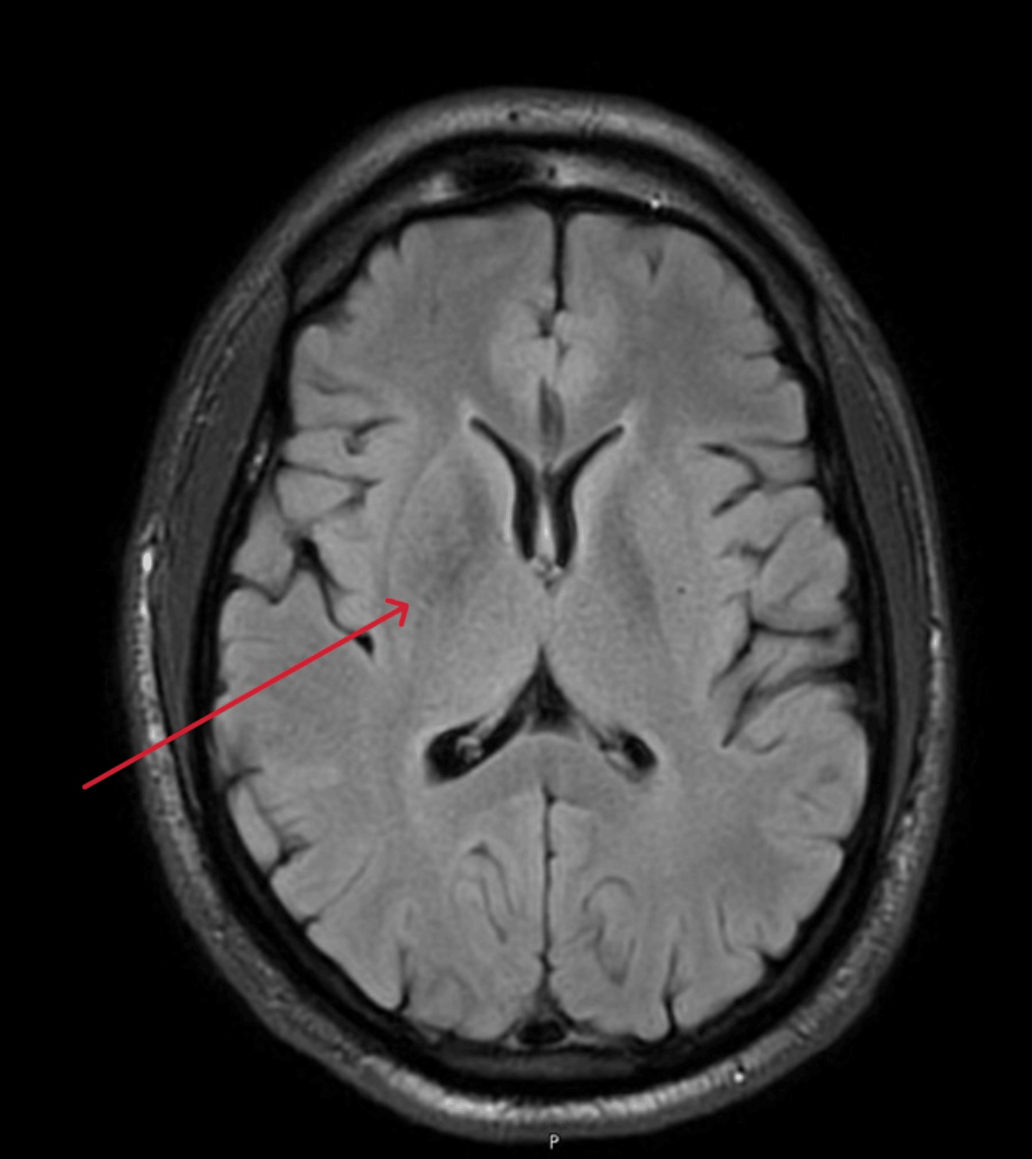
Magnetic resonance imaging/FLAIR revealing subtle hypointensity noted in the right basal ganglia (red arrow) FLAIR: fluid-attenuated inversion recovery

However, an MRI performed the following day revealed T1 hyperintensity in the right basal ganglia (Figure 2), with subtle T2/fluid-attenuated inversion recovery (FLAIR) hypointensity noted in the right basal ganglia (Figures 3, 4).

The patient was admitted with a suspected diagnosis of NHH, which was confirmed by MRI findings. He was started on an insulin sliding scale, followed by basal insulin glargine and oral sitagliptin/metformin (50/100 mg) two tablets per day, initiated the following day. He was also started on haloperidol 2 mg three times a day and clonazepam 1 mg once daily. He was also newly diagnosed with hypertension and started on amlodipine/valsartan 5/160 mg once daily. By day 3, the hemichorea improved, though some symptoms persisted. By day 4, with well-controlled blood sugar levels, the patient showed significant improvement, with complete resolution of leg movements and noticeable improvement in hand movements. The Internal Medicine team, including a diabetologist, managed him in coordination with the Neurology team. 

At discharge, he was clinically stable and discharged in good condition. His discharge medications included: amlodipine/valsartan 5 mg/160 mg once daily for blood pressure control, aspirin 100 mg daily, atorvastatin 40 mg daily, clonazepam 1 mg at bedtime, haloperidol 2 mg three times daily with a tapering plan, insulin glargine 18 units once daily, and sitagliptin/metformin 50/1000 mg sustained-release, two tablets once daily. He was referred for follow-up in both the Neurology and Medicine outpatient clinics to monitor his neurological recovery and optimize long-term diabetes and hypertension management.

## Discussion

NHH syndrome, also known as Chorea, Hyperglycemia, Basal Ganglia (C-H-BG) syndrome, is a rare but notable complication of poorly controlled diabetes mellitus or as the initial presentation of a newly diagnosed DM, typically presenting with a triad of hyperglycemia, involuntary muscle contractions (chorea or ballismus and dystonia), and basal ganglia hyperintensities on MRI [11,12]. Chorea, characterized by rapid, irregular, and involuntary limb movements, usually affects the distal limbs, while hemiballismus involves larger amplitude, more proximal movements. The disorder primarily occurs in older patients with long-standing type 2 diabetes, especially in women, and is associated with structural abnormalities in brain regions like the thalamus, caudate nucleus, and putamen [13]. Although other causes of chorea, such as infarcts, hemorrhages, or neoplasms, should be considered, NHH is a rare but important diagnosis, often presenting with unilateral involvement but occasionally affecting both sides. Prompt brain imaging is essential to distinguish this condition and guide appropriate treatment.

The pathophysiology of NHH remains unclear, but several mechanisms have been proposed. One theory suggests that hyperglycemia causes hyperviscosity, disrupting the blood-brain barrier, which allows inflammatory cells to infiltrate the basal ganglia and cause lesions [14,15]. Another hypothesis is that a potential reduction in the de novo synthesis of gamma-aminobutyric acid (GABA) by inhibitory GABAergic neurons may lead to unopposed activity of excitatory glutamatergic neurons. This imbalance can manifest clinically as abnormal movements, ranging from small-scale tremors to large-scale hemiballismus. Importantly, this phenomenon can occur in the absence of ischemia, potentially as a direct effect of hyperglycemia [7,16].

Additionally, the non-ketotic state in uncontrolled diabetes may contribute to a neuroexcitatory state, as ketone bodies, particularly β-hydroxybutyrate and acetoacetate, are known to have inhibitory effects on neuronal activity, potentially enhancing GABAergic tone and exerting anti-seizure properties. In the absence of ketosis, this protective modulation is lost, possibly predisposing to hyperexcitability and abnormal movements. This concept underlies the therapeutic use of ketogenic diets in some forms of epilepsy [17]. Despite these proposed mechanisms, the diagnosis and timely normalization of blood glucose typically lead to significant improvement in neurological symptoms, reinforcing the importance of glycemic control in managing NHH.

Diagnosis is based on three key components: clinical presentation with involuntary movements that diminish during sleep, laboratory findings indicating severe hyperglycemia and elevated HbA1c [18], and supportive imaging evidence. Neuroimaging plays a crucial role in diagnosing NHH, with MRI typically revealing hyperintense T1-weighted, hypointense T2-weighted, and CT hyperdensity lesions in the basal ganglia contralateral to the affected side [19]. Positron emission tomography (PET) can also demonstrate reduced glucose metabolism in the involved regions [20]. Imaging abnormalities often persist longer than clinical symptoms, sometimes lasting months or years despite glycemic control, underscoring the importance of early recognition and intervention [20].

The primary approach to managing NHH focuses on achieving optimal glycemic control, typically leading to symptom resolution. Studies suggest that patients treated with glucose regulation alone are more likely to complete recovery within a shorter time frame than those receiving both glycemic control and neuroleptic therapy [14]. While neuroleptics such as haloperidol may be beneficial for severe motor symptoms, they are generally tapered as blood sugar levels stabilize [19]. Sometimes, symptoms can take weeks or even months to fully resolve. For persistent chorea, additional medications like tetrabenazine or clonazepam may be considered, and in rare, refractory cases, surgical interventions such as deep brain stimulation or thalamotomy might be necessary [20].

This case highlights the importance of recognizing NHH as a rare yet reversible complication of uncontrolled diabetes. The characteristic clinical presentation and distinct neuroimaging findings aid in distinguishing NHH from other movement disorders. Our patient’s initial response to glycemic control underscores the importance of early intervention, though persistent symptoms highlight the variability in recovery time. Symptomatic treatment, such as neuroleptics, may provide partial relief, but ongoing monitoring and adjustments in management remain crucial. Given the potential for recurrence, strict long-term diabetes control is essential to reduce the risk of future episodes. This case further emphasizes the need for clinical vigilance, especially in patients with newly diagnosed diabetes presenting with acute-onset movement disorders.

## Conclusions

NHH is a rare but reversible neurological complication of poorly controlled diabetes, characterized by acute involuntary movements and striatal hyperintensities on MRI. In this case, a 41-year-old previously healthy man developed hemichorea affecting his left limbs, with MRI findings confirming NHH. His symptoms improved with insulin therapy and glycemic control, demonstrating the reversible nature of the condition. Early recognition and prompt metabolic correction are essential for symptom resolution and recurrence prevention, emphasizing the importance of long-term diabetes management in reducing future neurological complications.
